# Supplementary material for: Tubulin hyperacetylation drives HMGB1 nuclear exit via the ROS-PARP1 axis, leading to rotenone-induced G2/M arrest
Source: J Biol Chem. 2025 Sep 8;301(10):110695. doi: 10.1016/j.jbc.2025.110695 (PMC12538445; doi:10.1016/j.jbc.2025.110695)
Supplement: Supplementary legends_tables [file mmc1.docx]

**Fig. S1: Rotenone triggers HMGB1 nuclear release in PC12 cells but not N9 cells**

**(A)** Cell death analysis by SRB assay following indicated treatments of PC12 cells for 24h (n=3). **(B)** Representative immunofluorescence images showing HMGB1 (green) translocation after treatment with different concentration of Rotenone in PC12 cells (n=100 cells for each quantification scale bar=10 μm) (left panel), and the quantification is shown by manually counting cytoplasmic enriched HMGB1 (right panel). **(C)** Cell death analysis by SRB assay of N9 cells after indicated treatments with different concentrations of Rotenone for 24h (n=3). **(D)** Representative immunofluorescence image showing HMGB1 (green) translocation after treatment with different concentration of Rotenone in N9 cells (n=100 cells for each quantification scale bar=10 μm) (left panel), and the quantification is shown by manually counting cytoplasmic enriched HMGB1 (right panel). **(E)** Western blot analysis of PARylation level assayed in PC12 cells through probing with antibody against PAR, after treatment with rotenone. **F)** Representative immunofluorescence image showing HMGB1 (green) translocation after treatment with Rotenone alone or pre-treatment with PJ34 (10μM) in PC12 cells. (n=100 cells for each quantification, scale bar=10 μm), and the quantification is shown by manually counting cytoplasmic enriched HMGB1 (right panel). Data are presented as mean ± SD. Statistical significance was determined by one-way ANOVA with Tukey’s multiple comparison test and was indicated by ***p < 0.001.

**Fig. S2. HMGB1 interacts with PARP1 following rotenone treatment**

**(A)** Representative confocal images of co-localization of HMGB1 (green) with PARP1 (red) in cells treated with rotenone (5μM) or DMSO control for 4 h. DAPI (blue) indicates nuclear staining (scale bar=25 μm). **(B)** Co-immunoprecipitation analysis of HMGB1 and PARP1 in the whole cell extracts of cells following rotenone treatment for 4 h.

**Fig. S3.** **Rotenone induces G2/M arrest in SH-SY5Y and PC12 cells but not in N9 cells.**

**(A)** Representative flow cytometry histograms depicting DNA content in cells stained with propidium iodide (PI). Distinct peaks correspond to cells in the G0/G1 phase (first peak), S phase (intermediate region), and G2/M phase (second peak). SH-SY5Y cells were treated with rotenone for 24 h and the data is compared with DMSO control. **(B)** Immunoblot analysis of Cyclin B1 and Securin protein levels in the whole-cell extracts of SH-SY5Y cells 24 h post-rotenone treatment. β-Actin was used as a loading control. **(C-G)** Representative confocal images of SH-SY5Y cells stained with pH3S10 (red), HMGB1 (green) and DAPI (blue) following rotenone treatment for 2 h (C), 4 h (D), 6 h (E), 12 h (F) and 24 h (G). (scale bar=10 μm). **(H)** Quantification of pH3S10 positive cells indicating cells arrested at G2/M for each treatment. (n=100 cells for each quantification). **(I)** Representative confocal images of PC12 cells stained with pH3S10 (red), HMGB1 (green) and DAPI (blue) following rotenone treatment for 24 h (left panel) (n=100 cells for each quantification scale bar=25 μm) Quantification of pH3S10 positive cells indicating cells arrested at G2/M for each treatment (right panel). Enlarged images was cropped from the area marked by inset in merged images. **(J)** Representative confocal images of N9 cells stained with pH3S10 (red), HMGB1 (green) and DAPI (blue) following rotenone treatment for 24 h (left panel) (n=100 cells for each quantification scale bar =25 μm). Quantification of pH3S10 positive cells indicating cells arrested at G2/M for each treatment (right panel). Enlarged images was cropped from the area marked by inset in merged images. **(K)** Immunoblot analysis of Cyclin B1 protein levels in the whole-cell extracts of N9 cells 24 h post-rotenone treatment, 100ng/ml nocodazole treatment for 24h was used as a positive control. β-Actin was used as a loading control. Data are presented as mean ± SD. Statistical significance was determined by one-way ANOVA with Tukey’s multiple comparison test and was indicated by ***p < 0.001.

**Fig. S4. GA prevents rotenone induced G2/M cell cycle arrest in PC12 cells**

**(A)** Representative immunofluorescence images (Left panel) of PC12 cells stained with phospho-histone H3S10 (red) a G2/M phase marker, HMGB1 (green) and DAPI (blue) upon treatment with Rot 5µM and Rot 10µM or pre-incubation with HMGB1 cytosolic translocation inhibitor glycyrrhizic acid (500 µM). The percentage of cytoplasmic HMGB1 enriched cells and pH3S10 positive cells are plotted by manual counting (n=100 cells for each quantification scale bar=10 μm) (right panel). Data are presented as mean ± SD. Statistical significance was determined by one-way ANOVA with Tukey’s multiple comparison test and was indicated by ***p < 0.001.

**Fig. S5. si-αTAT1 prevents tubulin acetylation following rotenone treatment**

**(A)** Relative gene expression of αTAT1 for validation of knockdown by siαTAT1 was analysed through qRT-PCR using housekeeping control beta-2microglobulin (β2MG) utilizing two different oligos (n=3). **(B)** Representative immunofluorescence images stained with acetylated Tubulin (red) for the validation of αTAT1 KD in DMSO and Rot treated SH-SY5Y cells (scale bar=10 μm) (right panel). **(C)** Representative immunoblot images of acetylated tubulin protein level for validating the αTAT1 KD, Tubulin was used as a loading control. Data are presented as mean ± SD. Statistical significance was determined by Student’s T test and was indicated by ***p < 0.001.

**Fig. S6. The anti-oxidant NAC did not prevent rotenone-induced HMGB1 nuclear exit**

**(A)** Representative confocal images of HMGB1 (green) and DAPI (blue) in cells treated with rotenone for 24 h in the presence or absence of NAC (5mM). (scale bar=25 μm). **(B)** Immunoblot analysis of HMGB1 protein levels in the nuclear and cytosolic fraction of cells treated with rotenone for 24 h in the presence or absence of the NAC. GAPDH was used as cytosolic loading control and Lamin A/C was used as a nuclear loading control.

**Fig. S7. Rotenone triggers mtROS-induced DNA damage in PC12 cells**

**(A)** Representative live cell confocal images showing mitochondrial ROS levels after 4h Rotenone treatment in PC12 cells analysed through live staining with mitoSOX (red) and Hoechst (blue) (scale bar =10 μm). **(B)** Representative images of staining with phopsho-γH2AX (green), DAPI (blue) in rotenone treatment to show foci in PC12 cells, compared with DMSO treatment, (scale bar=10 μm) (left panel). Quantification was done by counting phopsho-γH2AX foci manually for atleast 100 cells (Right panel). Data are presented as mean ± SD. Statistical significance was determined by one-way ANOVA with Tukey’s multiple comparison test and was indicated by ***p < 0.001.

**Table S1. Primers used for cloning WT-HMGB1 and for site-directed mutagenesis**

| **Primer** | **Sequence (5’-3’)** |
| --- | --- |
| pCMV6-FLAG-HMGB1 WT Forward | ATGACAGCGATCGCCATGGGCAAAGGAGATC |
| pCMV6-FLAG-HMGB1 WT Reverse | GGCTAACTCGAGTTCATCATCATCATCTTCTTCTTC |
| HMGB1 K282930A Forward | GAGGAGCATGCGGCGGCGCACCCAGATG |
| HMGB1 K282930A Reverse | CCGACAAGTTTGCACAAAAAATGCATATGATG |
| HMGB1 K282930R Forward | GAGGAGCATAGGAGGAGGCACCCAGATG |
| HMGB1 K282930R Reverse | CCGACAAGTTTGCACAAAAAATGCATATGATG |
| HMGB1 K182-185A Forward | GAAAAAAGCGCGGCAGCGGCGGAAGAGGAG |
| HMGB1 K182-185A Reverse | AGCCTTGACAACTCCCTTTTTTGCTGCATCAGG |
| HMGB1 ∆NES Forward | ATGTGGAATAACACTGCTGC |
| HMGB1 ∆NES Reverse | ATGTTCTCCTTTGATTTTTGGGC |

| **Primer** | **Sequence (5’-3’)** |
| --- | --- |
| ATM Forward | AGTGTGCGAGACAAGAA |
| ATM Reverse | CAAAGTCTTGAGGAAGATAGTAAG |
| FEN1 Forward 1 | CCCAAAGGCCAGTCATC |
| FEN1 Reverse 1 | AGTAGCTCTTGATGTCATTCTC |
| XPA Forward 1 | CTGGAGGCATGGCTAAT |
| XPA Reverse 1 | TAACAGGTCCTGGTTGATG |
| GADD45A Forward 1 | TGGTGACGAATCCACATT |
| GADD45A Reverse 1 | TCCATGTAGCGACTTTCC |
| KLF4 Forward 1 | CCACCTTCTTCACCCC |
| KLF4 Reverse 1 | AAGGTTTCTCACCTGTGT |
| RAD23B Forward 1 | ACAAGTTCTGGAGGACATC |
| RAD23B Reverse 1 | AGGATTCTCTCGACCTATCT |
| Hu αTAT Forward Oligo 1 | GAAGCTCTTTGTACTGGATG |
| Hu αTAT Reverse Oligo 1 | CTGCAACATATACTGGAAGAG |
| Hu αTAT Forward Oligo 2 | CCAGTATATGTTGCAGAAGG |
| Hu αTAT 1 Reverse Oligo 2 | CTCCAGATTGTAGTGCTTATTC |

**Table S2. Primers use for qRT-PCR**
